# A Combination of the Cutaneous and Musculo-Cutaneous Methods of Amputation

**Published:** 1875-09

**Authors:** D. Hayes Agnew


A Combination of the Cutaneous and Musculo-Cutaneous Plans of Amputation.—*By D. Hayes Agnew, M.D.*—I have recently employed a new method of interpretation which appears to me to possess advantages worthy of notice. In raising the integument from the deep fascia, after the old plan of the skin-flap, whether circular or oval, a large number of its vessels are necessarily cut, which tends to jeopardize the vitality of the flap. Again, in this operation, the muscles being divided circularly, it is impossible to secure an accurate contact of the surfaces; irregular spaces or cavities between the muscles and flap will remain, in which the discharges collect and interfere with the healing.

If the musculo-cutaneous method be adopted, the skin, from its greater elasticity, retracting more than the muscles, leaves the latter hanging below, thus requiring that they be either abridged with the knife, or tucked and crowded in, so that the stump can be closed. The plan which obviates all these objections is the one to which I refer, and consists in making two oval cuts through the skin, down to the deep fascia, on opposite sides of the limb, raising the integument only a short distance—say three-quarters of an inch—and then applying the knife at the junction of the skin-flap and the deep fascia, and cutting the muscles obliquely back to the bone, or bones, as the case may be; or, if transfixation is preferred, thrust the knife through the angles of the tegumentary wound, and cut from within out. I find, however, the former the more convenient plan. The adjustment between the divided surfaces will be complete.

In the few cases in which I have performed the operation, the union has been quick and the form of the stump perfect. This method is adapted to any part of the fore-arm, arm, or thigh, and even the leg, as its details can be made practical on the posterior part of this portion of the lower extremity.—*Times.*

